# Advances in the Use of *N*-Acetylcysteine in Chronic Respiratory Diseases

**DOI:** 10.3390/antiox12091713

**Published:** 2023-09-02

**Authors:** Daniela Mokra, Juraj Mokry, Romana Barosova, Juliana Hanusrichterova

**Affiliations:** 1 Department of Physiology, Jessenius Faculty of Medicine in Martin, Comenius University in Bratislava, SK-03601 Martin, Slovakia; palova31@uniba.sk (R.B.); topercerova4@uniba.sk (J.H.); 2 Department of Pharmacology, Jessenius Faculty of Medicine in Martin, Comenius University in Bratislava, SK-03601 Martin, Slovakia; juraj.mokry@uniba.sk

**Keywords:** *N*-acetylcysteine, oxidative stress, chronic respiratory disorders, cystic fibrosis, COPD, asthma, IPF, silicosis

## Abstract

*N*-acetylcysteine (NAC) is widely used because of its mucolytic effects, taking part in the therapeutic protocols of cystic fibrosis. NAC is also administered as an antidote in acetaminophen (paracetamol) overdosing. Thanks to its wide antioxidative and anti-inflammatory effects, NAC may also be of benefit in other chronic inflammatory and fibrotizing respiratory diseases, such as chronic obstructive pulmonary disease, bronchial asthma, idiopathic lung fibrosis, or lung silicosis. In addition, NAC exerts low toxicity and rare adverse effects even in combination with other treatments, and it is cheap and easily accessible. This article brings a review of information on the mechanisms of inflammation and oxidative stress in selected chronic respiratory diseases and discusses the use of NAC in these disorders.

## 1. Introduction

In the background of the pathophysiology of the majority of respiratory diseases, inflammation with abundant production of pro-inflammatory cytokines and other bioactive substances including reactive oxygen and nitrogen species (RONS) can be found. For this reason, various antioxidants have been used in the treatment of these disorders, however, with variable therapeutic responses. *N*-acetylcysteine (NAC) is one of the most widely used therapeutic agents with antioxidant properties. Due to its mucolytic effects, it takes a part in the therapeutic protocols of cystic fibrosis (CF), and it is also used as an antidote in acetaminophen (paracetamol) overdosing [1,2,3]. In addition, wide anti-inflammatory and antioxidant effects favor the use of NAC in other chronic respiratory diseases, such as chronic obstructive pulmonary disease (COPD), bronchial asthma, idiopathic pulmonary fibrosis (IPF), or lung silicosis. Besides relatively low toxicity and rare adverse effects even in combination with other treatments, good accessibility and a low price represent the additional advantages of NAC.

The purpose of this article is to provide a review of the information on the mechanisms of inflammation and oxidative stress in selected chronic respiratory diseases and to critically discuss the use of NAC in these disorders. For this review, articles published in English from the PubMed database were used.

## 2. Pharmacological Effects of NAC

### 2.1. Pharmacokinetics and Pharmacodynamics of NAC

**Figure 1:**
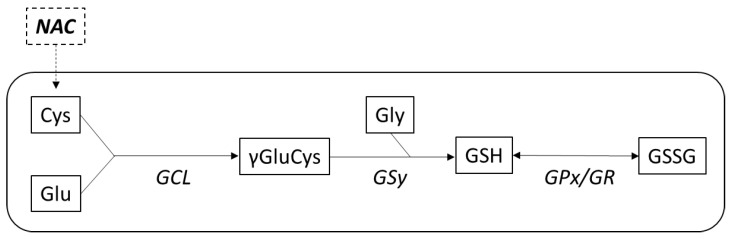
Entry of NAC into the cell and incorporation of cysteine in the cycle of glutathione (GSH) synthesis. Glutathione is synthetized in a two-step ATP-dependent pathway from amino acids glutamate (Glu), cysteine (Cys), and glycine (Gly) under a gradual catalysis by enzymes glutamate-cysteine ligase (γ-glutamyl-cysteine synthase) (GCL) and GSH synthase (GSy). GSH is oxidated by glutathione peroxidase (GPx) into oxidized glutathione (GSSG), which may be reduced by glutathione reductase (GR) from GSSG back to GSH.

NAC is a synthetic SH-group containing the *N*-acetyl derivative of the naturally occurring amino acid L-cysteine. It is the main substrate for the production of a potent antioxidant glutathione (GSH). After entering the cell, NAC is rapidly hydrolyzed into cysteine which is utilized for the synthesis of GSH [4] (Figure 1).

NAC is commonly used in oral, inhalational, or intravenous forms. For respiratory diseases, the maximum licensed daily dose for oral delivery is 600 mg; however, in numerous clinical studies, higher doses of NAC have been used [5,6,7,8]. NAC may reach higher concentrations in the airways after inhalational administration which may enhance its mucolytic effect. On the other hand, the wider use of nebulized NAC may be limited by the low intrinsic reducing activity and short half-life in the airways [9]. In oral administration, NAC is rapidly absorbed in the small intestine while the peak value of the plasma concentration is reached within 1–2 h after delivery [10]. Subsequently, NAC undergoes first-pass metabolism in the liver to cysteine, which is utilized for the synthesis of GSH. Due to changes during transport through the intestinal wall and metabolism in the liver, as well as due to rapid diffusion into the cells and conversion to GSH, the oral bioavailability of NAC is low (4–10%), with the lowest bioavailability shown for the slow release tablets [11,12]. After intravenous delivery, NAC skips the first-pass metabolism in the liver and changes in the intestinal wall which enables NAC to reach sufficient concentrations faster. Therefore, this way of administration is used, e.g., in paracetamol poisoning [13]. The elimination half-life of 600 mg of NAC given by intravenous infusion is about 2.3 h and the mean residence time is about 1.6 h, and after 12 h virtually no NAC is detected in the plasma [11].

### 2.2. Adverse Effects and Drug Interactions of NAC

NAC has an unpleasant smell and taste resembling rotten eggs that may limit its use. However, novel flavored effervescent formulations may solve this problem [14].

Adverse effects of NAC depend on the route of delivery [13]. Nevertheless, thanks to its low bioavailability, NAC has rather low toxicity. In the standard oral dosing of 600 mg/day for chronic use, NAC is well-tolerated [15] while several trials even showed no increase in the occurrence of side effects in the NAC-treated patients with chronic respiratory diseases compared to placebo-treated groups [7,8,16,17]. Although the side effects of oral NAC treatment may more frequently occur in high doses, they are relatively benign [15]. For oral administration, mild gastrointestinal symptoms such as dyspepsia, nausea, vomiting, or diarrhea are the most common adverse effects [16,17]. In high oral doses of NAC, dizziness, leg pain, leg edema, headache, palpitations, or worsening dyspnea may occur [18,19,20]. Rarely, symptoms related to allergic or anaphylactic reaction such as flushing, pruritus, erythema, bronchoconstriction, or hypotension may appear due to the release of histamine [21]. However, the occurrence of anaphylactoid adverse effects is more frequent in intravenous NAC delivery [22,23]. After inhalational administration of NAC, the local adverse effects from the airways and lungs such as cough, sore throat, pneumonia, etc., dominate [15,24]. Some authors found oral administration of NAC safer than inhalation [24], while other authors showed no differences in the occurrence of adverse effects in the comparison of the effects of three different doses (600 mg, 1800 mg, and 4800 mg) of intravenous NAC compared to controls [25]. However, because of the anticoagulant properties and inhibition of platelet aggregation, NAC should be used with caution in patients with bleeding disorders and anemia [26].

NAC potentiates the effect of nitroglycerine and the related drugs that may cause hypotension [10]. NAC may decrease the excretion of various drugs, including statins, digoxin, enalapril, bosentan, methotrexate, etc. The therapeutic effect of NAC may be reduced if combined with activated charcoal or antibiotics (tetracycline, oxytetracycline, and erythromycin) [27].

### 2.3. Mechanisms of Action of NAC

NAC exerts a wide range of actions. Details on the molecular mechanisms of the biological action of NAC have been recently published in several excellent review articles [1,2,3]. Therefore, we provide here only a short report of the basic information necessary for understanding the following chapters.

#### 2.3.1. Mucolytic Effect of NAC

**Figure 2:**
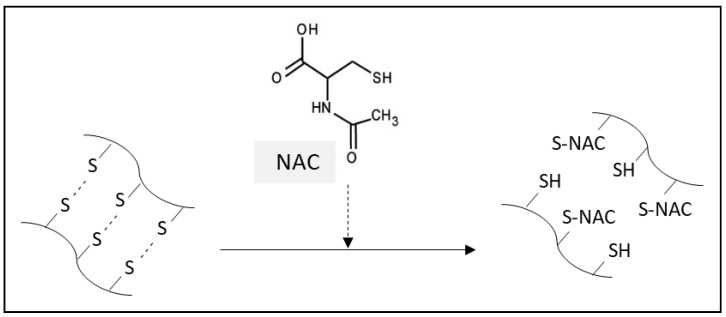
Scheme of mucolytic action of NAC.

Thanks to a potent mucolytic effect, NAC has been approved by the Food and Drug Administration (FDA) and the World Health Organization (WHO) as a treatment of choice in CF and may be also useful in other respiratory diseases with abundant production of thick and hardly removable mucus, such as COPD, bronchial asthma, or IPF [15]. The mucolytic action of NAC results from its ability to split the disulfide bonds of the high-molecular-weight glycoproteins of mucus (Figure 2).

Cystein-rich mucins undergo oxidation during inflammation that leads to the abnormal disulfide bridging between domains, stiffening of mucins, and slowing down of the mucociliary transport. The thickening of the mucus contributes to an obstructed airflow and predisposes to a higher risk of infections [28]. NAC possesses higher disulfide-reducing activity than cysteine or GSH due to its stronger nucleophilicity which decreases the viscosity of mucus and enables its removal from the airways [1,29].

In addition, breaking down the thiol proteins by NAC releases free thiols with antioxidant capacity representing an additional antioxidant mechanism of NAC [1].

#### 2.3.2. NAC as an Antidote

NAC has been used as an antidote for paracetamol (acetaminophen) intoxication since 1970 [30]. Paracetamol undergoes metabolic changes to *N*-acetyl-p-benzoquinone imine (NAPQI) which is detoxicated by GSH. In a shortage of GSH, NAPQI reacts with various proteins and nucleic acids and the generated by-products may subsequently trigger cell damage and/or an immune response, particularly in the liver and kidneys [31,32]. NAC as a precursor of cysteine restores mitochondrial GSH levels, scavenges reactive oxygen species (ROS)/peroxynitrite, enhances mitochondrial bioenergetics, and thus prevents cell damage [33]. However, exogenous *N*-acetylcysteine does not form significant amounts of conjugate with the reactive metabolite of acetaminophen in the rat in vivo but increases glutathione synthesis, thus providing more substrate for the detoxification of the reactive metabolite in the early phase of acetaminophen intoxication when the critical reaction with vital macromolecules occurs [34]. This finding suggests significant effects of NAC in acute paracetamol intoxication with short and fast action predominantly in the liver, compared to milder long-term antioxidant and anti-inflammatory effects in chronic diseases of the respiratory system. A similar action is presumably responsible for favorable effects of NAC in other types of poisonings induced, e.g., by heavy metals, herbicides, or mushrooms [2,35,36,37].

#### 2.3.3. Antioxidant Effects of NAC

The direct antioxidant action of NAC is related to the presence of the thiol (-SH) group and acetyl group linked to the amino group [13] which determine that NAC is able to react with various radical and non-radical RONS. Despite relatively low direct reactivity for hydrogen peroxide (H_2_O_2_) and superoxide (O_2_^•−^), NAC is highly effective in scavenging nitrogen dioxide (NO_2_) and hypohalous acids (HOX) and rapidly forms adducts with α,β-unsaturated aldehydes preventing their conjugation to proteins [1,28,38]. The indirect antioxidant effects of NAC mainly result from a replenishment of GSH through the cellular delivery of cysteine (formed by NAC deacetylation) [39] together with glutamate and glycine which are essential for the synthesis of GSH (Figure 1). GSH as a direct antioxidant regulates the oxidative status in the cells and simultaneously acts as a substrate for several antioxidant systems, e.g., glutathione reductase, glutaredoxin, glutathione peroxidase, or peroxiredoxin [40]. Thioredoxin and GSH systems are dependent on the generation of energy in mitochondria and, vice versa, mitochondria are responsible for maintaining redox homeostasis and producing ATP depending on the thioredoxin and GSH systems [41,42].

In addition, NAC enhances the expression of the nuclear factor erythroid-2-related factor 2 (Nrf2), a transcription factor which acts as a regulator of antioxidant and cytoprotective enzymes [42,43]. In oxidative stress, Nrf2 binds to a DNA promoter of the nucleus named the antioxidant responsive element (ARE) and initiates the transcription of antioxidative genes promoting GSH biosynthesis or maintaining the GSH redox status. Administration of NAC up-regulates Nrf2 leading to, e.g., improved cerebral mitochondrial function, decreased oxidative and nitrosative stress, and reduced neuroinflammation [42]. Binding through the ARE, NAC also stimulates anti-inflammatory mechanisms via influencing hemoxygenase-1 (HO-1) or nicotinamide adenine dinucleotide phosphate (NADPH)-dehydrogenase-1 [44].

As the levels of GSH decline with age [45], positive effects of supplementation with NAC in combination with glycine on changes associated with aging have been successfully tested in clinical trials [46,47]. Because GSH concentrations also decrease in various disorders with abundant oxidative stress [48], NAC has been used in a variety of respiratory diseases, including COPD [49], IPF [19], lung silicosis [50], COVID-19 [51,52], or obstructive sleep apnea [53], as well as in multidrug-resistant infections [54], cardiovascular diseases [55], type 2 diabetes [56], chronic nephropathy [57], or infertility due to polycystic ovaria [58].

#### 2.3.4. Anti-Inflammatory Effects of NAC

Oxidative stress and inflammation are very closely linked. Oxidative stress induces the expression of nuclear factor (NF)-κB which subsequently increases the synthesis of pro-inflammatory cytokines, such as tumor necrosis factor (TNF)α, interleukins (IL)-1β, -6, -8, etc. [3,59]. NF-κB may be also directly activated by TNFα which acts as a pluripotent activator of inflammation; however, NAC has a potential to mitigate these TNFα-mediated effects [60,61,62].

Moreover, NAC modulates other pro-inflammatory mechanisms, including cyclooxygenase (COX)-2, matrix metalloproteinases (MMP)-3 and -4, or the expression of the intercellular adhesion molecule (ICAM)-1, and inhibits the phosphorylation of p38 mitogen-activated protein kinase (MAPK) by decreasing the intracellular concentration of H_2_O_2_ and restoring the redox balance [63].

#### 2.3.5. Anti-Fibrotic Effects of NAC

Discovering a significant role of oxidative stress in the pathogenesis of lung fibrotizing diseases such as IPF and silicosis [64,65,66,67] has suggested that restoring the redox balance may be of benefit in these diseases, as well. In in vitro studies, NAC decreased the alveolar epithelial–mesenchymal transition (EMT) and/or diminished production of fibrosis markers, such as collagen, α-smooth muscle actin (SMA), or fibronectin [68,69,70].

Comparably in in vivo experiments, NAC inhibited several profibrotic mechanisms in murine models of bleomycin-induced [71,72,73,74,75] and silica-induced [76,77] lung fibrosis. However, the relevance of the anti-fibrotic effects of NAC has not been clearly confirmed yet in the clinical studies in patients with IPF [5,19,78,79,80] or with lung silicosis [50,81].

#### 2.3.6. Cytoprotective Effects of NAC

Crosslinking the cysteine disulfide molecules, NAC stabilizes the proteins while this modification is versatile and reversible [82]. In addition, NAC stabilizes DNA by mechanisms of DNA repair/protection, including the modulation of DNA repair, inhibition of genotoxicity and cell transformation, regulation of cell survival and apoptosis, inhibition of progression to malignancy, etc., and thereby exerts cancer-preventing effects [83,84,85].

**Figure 3:**
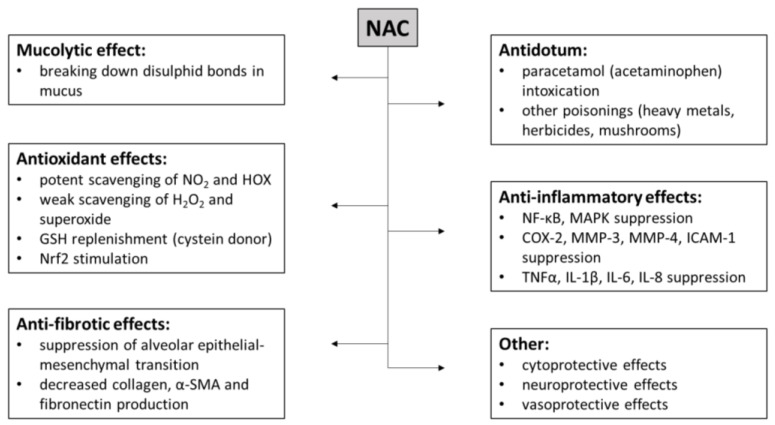
Biological effects of NAC. Abbreviations: COX-2: cyclooxygenase-2, GSH: glutathione, H_2_O_2_: hydrogen peroxide, HOX: hypohalous acids, ICAM-1: intercellular adhesion molecule-1, IL: interleukin, MAPK: mitogen-activated protein kinase, MMP: matrix metalloproteinase, NAC: *N*-acetylcysteine, NF-κB: nuclear factor kappa B, NO_2_: nitrogen dioxide, Nrf2: nuclear factor erythroid-2-related factor 2, α-SMA: alpha-smooth muscle actin, TNFα: tumor necrosis factor alpha.

A summary of the most important biological effects of NAC is provided in Figure 3.

## 3. NAC in Chronic Respiratory Diseases

### 3.1. NAC in CF

#### 3.1.1. Pathophysiology and Treatment of CF

CF is an autosomal recessive disease caused by a genetic mutation of the gene encoding the cystic fibrosis transmembrane conductance regulator (CFTR). The CFTR acts as a cyclic adenosine monophosphate (cAMP) anion channel that conducts chloride and bicarbonate at the apical membrane of epithelial cells and controls water and ion transport [86]. In the airways, bicarbonate release is fundamental for the proper unfolding of mucins and local defense; however, impaired bicarbonate transport also contributes to problems in the pancreas, intestine, reproductive organs, salivary glands, etc. [87]. Production of viscous and hardly removable mucus in the airways predisposes to repetitive infection that may result in lung damage and fibrosis [88]. In addition, the biophysical properties of mucus worsen under the influence of oxidation arising from airway neutrophilic inflammation or environmental exposure leading to increased mucin polymer crosslinks and stiffening of the airway mucus gels [89]. Airway inflammation in CF is characterized by abundant neutrophil recruitment and by an imbalance between pro- and anti-inflammatory factors [90,91]. Oxidative stress due to infections is further aggravated by the malabsorption of dietary antioxidants in the intestine and the inability of cells with the CFTR mutation to efflux GSH [91].

As mentioned before, administration of NAC increases a pool of cysteine-breaking sulfhydryl bridges among mucus glycoproteins and thereby enhances mucus clearance from the airways [29]. NAC may be useful as a donor of cysteine for the production of GSH as GSH exocytosis is likely dysregulated because of dependence on the above-mentioned CFTR channel [92]. In addition, besides the proposed indirect effect on the CFTR via antioxidant and anti-inflammatory action, NAC may restore defective autophagy caused by decreased levels of beclin-1 which is involved in autophagosome formation and lung inflammation in CF [93].

In addition to NAC, other drug therapy including CFTR modulators such as ivacaftor, extensive physiotherapy, and nutritional support are used in the management of CF [94].

#### 3.1.2. NAC in In Vitro Studies of CF

There are numerous in vitro studies demonstrating the positive effects of NAC on selected types of cells. For instance, the incubation of A549 cells with NAC reduced the concentration of a deleted in malignant brain tumors 1 protein (DMBT1), a protein acting on inflammation, angiogenesis, and epithelial differentiation, and altered the ciliary motility [95]. Besides restoring the redox imbalance and airway inflammation in CF, NAC may prevent and eliminate biofilms resulting from airway infections, particularly of *Pseudomonas aeruginosa* [91,96]. The potential of the combination of NAC and antibiotics in CF has been intensively studied in in vitro conditions [97,98].

#### 3.1.3. NAC in Animal Studies of CF

There are just a few studies demonstrating the effects of NAC in animal models of CF. One of them compared NAC effects with other mucolytics and found that dithiothreitol and P3001 exhibited superior activities in reducing the viscoelasticity in vitro than NAC. The in vivo part of the experiments showed a significant decrease in the lung mucus burden after P3001 in βENaC-overexpressing mice, while no effect was found for NAC [99].

#### 3.1.4. NAC in Clinical Studies of CF

In patients with CF, NAC decreased the migration of neutrophils into the lungs, the release of elastase-rich granules, and the production of IL-8 by airway neutrophils [6]. In a phase II clinical study evaluating the safety and efficacy of an oral low dose (700 mg daily) and high dose (2800 mg daily) during 12 weeks of therapy in patients with CF, high-dose NAC was well-tolerated and showed a tendency to increase the extracellular GSH; however, it did not change the clinical or inflammatory markers [100]. Comparably, in another study, the administration of aerosolized NAC was well-tolerated, with a decrease in the sputum viscoelasticity observed in a dose-dependent manner [101].

In more recent clinical trials, NAC slightly enhanced the lung functions, increased the plasma levels of ascorbic acid, and decreased the levels of the oxidized form of ascorbic acid [102], or it maintained the lung function without the change in biomarkers of neutrophilic inflammation [103]. Similarly, in a small group of NAC-treated patients with CF, aerosolized NAC was rapidly cleared from the lungs and did not influence the sputum biophysical properties [99].

### 3.2. NAC in COPD

#### 3.2.1. Pathophysiology and Treatment of COPD

Oxidative stress is an important driving mechanism of COPD via the initiation of chronic inflammation associated with stimulated mucus secretion, induction of cell senescence and damaged autophagy, decreased DNA repair, enhanced autoimmunity, and impaired response to corticosteroid treatment [104]. The complex pathophysiology of COPD leads to general symptoms such as dyspnea, cough, and sputum production, while acute exacerbations result from respiratory infections or exposure to environmental factors. Treatment of COPD covers both pharmacologic approaches (long-acting β2-agonists, long-acting muscarinic antagonists, inhaled corticosteroids, and mucolytics) and non-pharmacologic approaches (smoking cessation and pulmonary rehabilitation) [105].

Oxidative stress in COPD originates from exogenous factors, such as cigarette smoking and air pollution, and/or from endogenous factors including ROS produced by activated inflammatory cells, particularly neutrophils and macrophages, in the lungs. Simultaneously with increased oxidative stress, endogenous antioxidants such as Nrf2-dependent antioxidants and GSH decrease, and the situation may be worsened by a low intake of dietary antioxidants [104,106]. Generated ROS enhance the activation of intracellular signaling pathways including the expression of NF-κB, p38 MAPK, Jun-*N*-terminal kinase (JNK), PI3 kinase, protein tyrosine phosphatases, etc., leading to the synthesis of various pro-inflammatory mediators (cytokines, chemokines, and growth factors) [107]. The exposure to inhaled irritants activates epithelial cells and macrophages to produce chemotactic factors engaging inflammatory cells into the lungs, e.g., the CC-chemokine ligand (CCL) 2 attracting monocytes; CXC-chemokine ligand (CXCL) 1 and CXCL8 attracting neutrophils and monocytes; CXCL9, -10, and -11 which act on the CXCR3 attracting T helper (Th) 1 cells and type 1 cytotoxic T (Tc1) cells; or CCL5 attracting eosinophils [107,108]. The activated cells generate pro-inflammatory cytokines such as TNFα, IL-1β, and IL-6 which amplify inflammation as well as proteases including MMP-9 which cause elastin degradation and emphysema. In addition, epithelial cells and macrophages produce the transforming growth factor (TGF)-β and fibroblast growth factors (FGFs) which stimulate fibroblast proliferation and cause fibrosis in the small airways. Mucus hypersecretion, a typical sign of COPD, is stimulated by the epithelial growth factor (EGF), TGF-α, and neutrophil elastase [107,108].

COPD is also linked to accelerated lung aging with the accumulation of senescent cells generating various inflammatory mediators and ROS [104,109]. In addition, there is an increasing number of dysfunctional mitochondria which were not eliminated due to a defect in mitophagy but produce large amounts of ROS [110].

Cigarette smoking is a main risk factor of COPD because it brings a large number of potentially noxious substances into the lungs. In addition, cigarette smoke decreases the activity of the CFTR and mucociliary transport contributing to the pathophysiology of COPD [111].

#### 3.2.2. NAC in In Vitro Studies of COPD

Several in vitro studies brought new information on the pathomechanisms of COPD or the effects of NAC. For instance, one of the older in vitro studies helped to identify the cigarette smoke-induced oxidative changes in the lung neutrophils and epithelial cells and evaluated the potential of NAC to alleviate these changes by increasing GSH [112]. In addition, NAC increased the activity of the multidrug-resistance-associated protein (MRP)1 which protects against toxic compounds and oxidative stress as well as smoke-induced disease progression [113]. An in vitro analysis of peripheral blood neutrophils from COPD patients showed that NAC pretreatment decreased the neutrophil chemotaxis, elastase release, and production of ROS and increased the thiol levels [114]. In addition, NAC protects from oxidative stress via Nrf2 signaling as demonstrated in alveolar type II cells [115]. In a recent in vitro study, the cigarette smoke-induced CFTR dysfunction impaired the phagocytosis in the alveolar macrophages and the cigarette smoke also inhibited the mitochondrial respiration while inducing glycolysis and the generation of ROS. However, these effects were mitigated by NAC [116].

#### 3.2.3. NAC in Animal Studies of COPD

In a rat model of COPD, cigarette smoke exposure for 3.5 months elevated IL-4 and MMP-12, decreased interferon (IFN)-γ, increased the IL-4/IFN-γ ratio and MMP-12/tissue inhibitor of the metalloproteinases (TIMP-1) ratio, and worsened lung functions. Treatment with NAC enhanced lung functions, increased IFN-γ, and decreased the IL-4/IFN-γ ratio while the MMP-12/TIMP-1 ratio remained unchanged [117]. In another cigarette smoke-induced model of COPD in rats, NAC attenuated lung damage, emphysema, and alveolar septal cell apoptosis by partly reversing a decrease in vascular endothelial growth factor (VEGF) secretion and VEGFR2 protein expression [118]. In an ozone-induced COPD murine model, preventive use of NAC decreased the count of macrophages in the bronchoalveolar lavage fluid (BALF) and reduced the airway smooth muscle mass, while therapeutic NAC administration reversed the airway hyperreactivity and reduced the airway smooth muscle mass and the number of apoptotic cells [119]. In a rat model of COPD, NAC treatment enhanced pulmonary functions, decreased COPD-induced increases in TNFα and IL-6, reduced the inflammatory infiltration of the lung, reduced the tissue injury and destruction of the alveolar septum, decreased the thickness of the bronchiolar wall, and reduced the collagen and α-SMA levels [120]. In addition, NAC decreased the expression of the von Willebrand factor gene, one of the target genes for COPD, and reduced the p38 MAPK phosphorylation that resulted in the inhibition of the EMT and relieved the pulmonary fibrosis [120].

#### 3.2.4. NAC in Clinical Studies of COPD

Although the mucolytic and antioxidant effects of NAC may be of great value in COPD, beneficial effects of NAC have not been clearly confirmed in the clinical studies, probably due to insufficient doses, short time of observation, inactivation of NAC by oxidative stress, and/or the choice of inadequate outcome parameters [7,104]. The addition of NAC at a dose of 600 mg twice daily to treatment with corticosteroids and bronchodilators did not modify the outcome in acute exacerbations of COPD [121]. In a randomized placebo-controlled study performed in 50 centers, 523 patients with COPD were given 600 mg daily of NAC or placebo. However, observation for 3 years failed to show any prevention of the deterioration in lung functions or prevention of exacerbations [122]. In another randomized controlled trial (NCT00476736), NAC treatment (1200 mg/day) improved physical performance, probably due to a reduction in air trapping [123]. In the HIACE randomized controlled trial (NCT01136239), 1-year treatment with high-dose NAC (600 mg bid or placebo after a 4-week run-in) resulted in enhanced small airways function and a lower exacerbation frequency in patients with stable COPD [7]. A trial by Zheng et al. showed that the long-term use of NAC 600 mg twice daily may prevent exacerbations, particularly in patients with moderate-to-severe COPD [8]. In another RCT (NCT01599884), patients with COPD and chronic bronchitis received oral NAC (1800 mg) or a placebo twice daily for 8 weeks in addition to their usual respiratory medications. However, the study was prematurely terminated because of finding no clinical benefit [20]. A similar absence of improvement has been recently demonstrated in patients with COPD and chronic bronchitis treated with 900 mg NAC twice daily for 3 months [124]. However, an analysis of the peripheral blood of patients with COPD showed that oral administration of NAC (dose of 400 mg, 3 times a day, for 6 months) increased a proportion of the Treg in the CD4^+^ T cells and decreased the Th17/Treg ratio, whereas the regulation of the Th17/Treg balance was mediated via the hypoxia-inducible factor (HIF)-1α pathway [125]. NAC showed its antioxidant and anti-inflammatory effects also in the ex vivo models of COPD exacerbation [126,127].

### 3.3. NAC in Bronchial Asthma

#### 3.3.1. Pathophysiology and Treatment of Asthma

Asthma is a heterogenous disorder which appears in several endotypes defined according to underlying pathophysiological mechanisms and several phenotypes defined by clinical characteristics [128,129]. The reversible airway obstruction and production of mucus which are typical for asthma are accompanied with chronic inflammation. The type of inflammation, i.e., eosinophilic, neutrophilic, or mixed granulocytic inflammation, represents the main criterium to assign the patient into the asthma endotype with different responsiveness to treatment [128,129]. While eosinophilic asthma is effectively treated with inhaled/oral corticosteroids, bronchodilators, xanthine derivatives, biological treatments, etc., neutrophilic asthma does not respond to corticosteroids; therefore, macrolide antibiotics and biological treatments are recommended together with bronchodilators and xanthine derivatives [128].

Regardless of the type of predominant inflammation, activated cells produce significant portions of ROS [130]. In addition, the redox imbalance in asthma is aggravated by decreased activity of endogenous antioxidants, i.e., superoxide dismutase (SOD), catalase, glutathione peroxidases, heme oxygenase-1, thioredoxins, peroxiredoxins, glutaredoxins, etc. [131]. These findings have inspired the use of various dietary antioxidants to enhance the clinical status of patients with asthma [132,133,134].

#### 3.3.2. NAC in In Vitro Studies of Asthma

In human airway smooth muscle cells, NAC inhibited the IL-1β-induced ROS production and eotaxin and monocyte chemotactic protein (MCP)-1 expression via decreased activation of p38 MAPK [135]. The positive effects of NAC were also observed in human eosinophils where NAC suppressed the generation of ROS, augmented the eosinophil content of reduced GSH, and decreased the release of the eosinophil cationic protein (ECP) [136]. In THP-1 cells, NAC inhibited ROS production induced by thymic stromal lymphopoietin (TSLP), a Th2-like cytokine involved in asthma pathogenesis [137].

#### 3.3.3. NAC in Animal Studies of Asthma

In a rat model of asthma, oral pretreatment with NAC reduced airway hyperresponsiveness, decreased lipid peroxidation and oxidized glutathione levels, and lowered the TNFα, inducible nitric oxide (NO) synthase, ICAM-1, and mucin MUC5AC expressions [138]. In a murine model of the steroid-resistant acute exacerbation of asthma, intraperitoneal NAC delivered 30 min before and 12 h after each ovalbumin (OVA) challenge reduced the airway hyperresponsiveness and decreased the neutrophil and eosinophil counts and inflammatory cytokines IL-13 and IL-5 in the BALF [139]. In addition, NAC exerted its potential in reducing airway inflammation, mitochondrial damage, and eosinophil extracellular traps release as it reduced eosinophil peroxidase (EPO), goblet cells hyperplasia, pro-inflammatory cytokines production, NF-κB p65 content, and lung oxidative stress and enhanced the mitochondrial energy metabolism in the OVA-challenged mice [140]. In a murine model of allergic asthma, NAC decreased OVA-induced airway hyperresponsiveness and inflammation by modulating the expression of claudin 18, a tight junctional protein expressed in airway epithelial cells [141]. In a murine model of obesity-associated asthma, NAC treatment decreased malondialdehyde (MDA) and elevated GSH and suppressed NF-κB activation as demonstrated by a decline in inhibitor κB kinase (IKK)-β and NF-κB-P65 and elevation in inhibitor (I)κB-α [142]. In a chemical-induced asthma model, NAC reduced the toluene diisocyanate-induced airway hyperresponsiveness and metaplasia of goblet cells, decreased the lung infiltration by neutrophils and eosinophils, lowered the production of IL-4 and IL-5, and attenuated the redox imbalance [143].

#### 3.3.4. NAC in Clinical Studies of Asthma

Despite promising findings from the above-mentioned preclinical research, the clinical studies evaluating the effects of NAC in asthma are rare and the results have been rather discouraging [144,145], probably due to the relative in vivo instability of NAC [146].

### 3.4. NAC in IPF

#### 3.4.1. Pathophysiology and Treatment of IPF

IPF is a progressive disorder presenting as interstitial pneumonia and fibrosis with the absence of an identifiable etiology which occurs mainly in elderly people [147]. Among multiple risk factors for IPF, aging and exposure to cigarette smoke have been identified as the main ones [148]. The features of IPF include the aberrant activation of alveolar epithelial cells, probably under the influence of environmental factors such as cigarette smoke or air pollution, and the accumulation of fibroblasts and myofibroblasts producing excessive amounts of the extracellular matrix [149]. Aging impairs both innate and adaptive immune mechanisms including cellular defense against pathogens and environmental insults, such as cigarette smoke. Lung damage and tissue remodeling also depend on the cell apoptosis imbalance and exhaustion of stem cell populations in the lung. The loss of homeostasis between epithelial and mesenchymal cells and increased resistance of myofibroblasts to apoptosis may then result in the abundant fibroblast activation and accumulation of the extracellular matrix leading to pulmonary fibrosis [148,150]. The above-mentioned changes are associated with a significant oxidative-antioxidant imbalance and GSH deficiency [151,152,153,154]. Oxidative stress in pulmonary fibrosis is associated mainly with the NF-κB signaling pathway, the Kelch-like ECH-associated protein 1 (Keap1)/Nrf2/ARE pathway, and the NADPH oxidase (NOX)4-Nrf2 signaling cascade [155]. Oxidative stress is linked to the biology of aging, influencing DNA damage responses and mitochondrial dysfunction. However, the increased production of ROS also participates in fibrotic processes, including the polarization and immunosenescence of macrophages, apoptosis and senescence of alveolar epithelial cells, differentiation and senescence of myofibroblasts, and alterations in the acellular extracellular matrix [152].

In the treatment of IPF, the oral anti-fibrotic drugs pirfenidone and nintedanib are used which can mitigate the symptoms and slow the disease progression; however, other novel approaches including biological treatments have been tested [156]. With regard to the relevant role of oxidative stress in the pathophysiology of pulmonary fibrosis, various antioxidants including NAC have been used in animal models or in clinical trials and the results have been discussed in numerous review articles [3,154,157].

#### 3.4.2. NAC in In Vitro Studies of IPF

In vitro studies showed that NAC prevents GSH depletion in various cells including fibroblasts and inhibits collagen production and the alveolar EMT [68,69]. NAC also diminished the production of TGF-β1-induced fibronectin and VEGF and the expression of α-SMA in human lung fibroblasts [70]. In addition, NAC suppressed the generation of various pro-inflammatory and pro-fibrotic mediators in epithelial cells, macrophages, and lymphocytes isolated from patients with IPF [158,159].

#### 3.4.3. NAC in Animal Studies of IPF

The anti-fibrotic effects of NAC were also demonstrated in several in vivo studies. For instance, the oral pretreatment of mice with NAC given daily for one week prior to intratracheal instillation of bleomycin decreased the collagen content in the lung [160]. Comparably in rats, NAC pretreatment decreased lung collagen and inflammatory cells and increased the total GSH levels in the lung but failed to decrease lung edema formation and protein and cell accumulation in the BALF [72]. A later study by this team showed that pretreatment with NAC reduced the lung fibrotic area, mitigated bleomycin-induced increases in lung TNFα and myeloperoxidase (MPO) activity, and decreased the numbers of mucus secretory cells in the airway epithelium and MUC5ac expression, indicating that oral NAC improved pulmonary lesions and alleviated bleomycin-induced mucus hypersecretion [73].

In another study, the oral administration of NAC (3 mmol/kg) in rats which started one day before intratracheal bleomycin instillation prevented the development of pulmonary fibrosis as indicated by lower lung hydroxyproline content, reduced the depletion of GSH peroxidase, and prevented bleomycin-induced increases in the MPO activity and elevations of NO and MDA in the lung [161]. Oral treatment with NAC (490 mg/kg b.w.) given to bleomycin-injured rats from day 0 reversed the lysyl oxidase activity, which is essential for collagen deposition, to normal levels; elevated the levels of lung GSH; partially attenuated pulmonary fibrosis; and inhibited TGF-β(1) and α-SMA expressions [75].

Intraperitoneally given NAC (300 mg/kg/day) decreased lung edema formation, the levels of proteins and lactate dehydrogenase, and the counts of neutrophils and macrophages in the BALF; lowered the activity of lung MPO and lung GSH/oxidized GSH (GSSG); suppressed the bleomycin-induced activation of lung NF-κB and decreased the levels of early pro-inflammatory cytokines (TNFα, IL-1β, IL-6, and macrophage inflammatory protein (MIP)-2); decreased collagen deposition; and improved the lung histological score [74]. In the bleomycin-exposed mice, NAC (20 mg/kg) in combination with an iron chelator deferoxamine (30 mg/kg) administered 60 days after bleomycin exposure almost completely reversed a lung injury and decreased the lactate dehydrogenase (LDH) activity and MDA levels, while NAC reduced the bleomycin-induced increases in the LDH and the total cell and neutrophil counts [162]. In rats with bleomycin-induced pulmonary fibrosis, intragastric NAC treatment (250 mg/kg) improved the lung appearance, decreased the MDA content, increased the SOD content, and lowered the degrees of alveolar inflammatory cell infiltration and fibrosis, but even better were the results for the combination of NAC with intraperitoneal administration of the free radical scavenger edaravone (6 mg/kg) [163]. The effects of intragastric NAC (4 mg/kg, 3 times a day) on air-sacculitis and the expressions of TGF-β1, TNFα, and the platelet-derived growth factor (PDGF) were comparable with the effects of anti-fibrotic agent pirfenidone and corticosteroid prednisone, while the lung fibrosis score and caveolin-1 were more effectively influenced by pirfenidone [164]. In a murine model, NAC treatment decreased the pulmonary ROS levels, enhanced the survival rate, and ameliorated premature senescence and pulmonary dysfunction by down-regulating TGF-β1/IL-11/MEK/ERK signaling [165].

#### 3.4.4. NAC in Clinical Studies of IPF

Despite encouraging data from preclinical studies, the results from clinical trials have been rather heterogenous. Clinical testing of NAC efficacy in pulmonary fibrosis started in the 1990s when several open-label studies demonstrated an increase in total GSH levels and an improvement in pulmonary function tests in patients with various types of pulmonary fibrosis after short-term NAC treatment [25,166,167]. However, in the patients with early stage IPF (*n* = 38) who received 352.4 mg of NAC by inhalation twice daily for 48 weeks, there were no significant overall differences in the change in forced vital capacity (FVC), although a higher stability of FVC was observed in two subsets of patients [168]. In a continuation of that study, the authors found that NAC therapy was effective in patients with mild-to-moderate IPF and NAC was more beneficial in those patients who had greater declines in FVC before the initiation of therapy [169]. In a retrospective observational study, inhaled NAC therapy caused a significant inverse correlation between the mean change in the GSSG level and FVC, suggesting that the patients with more severe oxidative stress may be good responders to inhaled NAC therapy, as GSH replenished by NAC inhalation can partially reverse an oxidant–antioxidant imbalance [170]. On the contrary, in the PANTHER-IPF trial (NCT00650091) evaluating the effectiveness of NAC monotherapy, no significant benefit of NAC on FVC was demonstrated [19,171]. However, a post hoc exploratory analysis of the subjects enrolled in the PANTHER-IPF clinical trial determining specific polymorphisms in the toll-interacting protein (TOLLIP) and MUC5B genes showed that NAC improved the prognosis in genetically predisposed individuals carrying the *rs3750920* (TOLLIP) TT genotype [78]. In the ongoing genotype-stratified clinical trial (the PRECISIONS trial, NCT04300920), the effects of NAC plus standard care were evaluated in IPF patients who have the TOLLIP *rs3750920* TT genotype, and the end of the study is estimated to be in 2025. Another ongoing trial is a phase I/II open-label pilot study (NCT03720483) which has the ambition to investigate the safety and tolerability of inhaled NAC in patients with IPF. After a withdrawal due to COVID-19, the study may finish at the end of 2023.

The effects of NAC have been also tested in combinations with other potentially effective treatments. For instance, the effectiveness of NAC (oral dose of 600 mg three times daily over one year) for IPF was evaluated in the IFIGENIA trial (NCT00639496) where three-drug therapy with NAC added to prednisone and azathioprine was more effective than the standard therapy with prednisone plus azathioprine [5]. At the 12-month follow-up observation, the combined delivery of inhaled NAC and oral pirfenidone reduced the rate of annual FVC decline and improved the progression-free survival in patients with advanced IPF [80]. On the contrary, a phase II trial (the PANORAMA study, NCT02707640) showed no benefit of NAC addition on the tolerability profile of pirfenidone in IPF [79]. Similarly, in a phase III clinical trial (UMIN000015508) evaluating the efficacy and safety of pirfenidone combined with inhaled NAC for IPF, no clinical improvement was observed for the combined treatment [172]. In another study, NAC combined with pirfenidone and NAC combined with budesonide were both effective in the treatment of IPF. However, a recent study has shown that the addition of NAC (600 mg, 3 times a day) to pirfenidone led to a lower incidence of adverse reactions and improved the quality of life and survival compared to a control group treated with a combination of NAC and budesonide, although both regimens equally ameliorated the inflammatory response and improved lung function [173].

### 3.5. NAC in Lung Silicosis

#### 3.5.1. Pathophysiology and Treatment of Lung Silicosis

A triggering factor for lung silicosis is the long-term inhalation of silica particles, usually as a professional burden [174]. The contact of lung cells with silica crystals leads to the generation of ROS due to the piezoelectric properties of the silica crystals [175] while additional amounts of ROS are produced due to silica-induced inflammation. After silica particles are inhaled, they are recognized by the surface receptors of the alveolar macrophages and overwhelmed by these cells. Nevertheless, the lysosomal system cannot break down these particles which leads to the damage of the lysosomal membrane and the release of lysosomal enzymes. The process continues in a vicious cycle when released silica is engulfed by other alveolar macrophages [176]. Enzymes liberated from the lysosomes, increased concentrations of ROS, and the activation of surface receptors on the immune cells activate various pro-inflammatory pathways, including NF-κB. These factors contribute to the activation of the NLR family pyrin domain-containing 3 (NLRP3) inflammasome resulting in mitochondrial or cell apoptosis, pyroptosis, a highly pro-inflammatory type of cell death, and stimulation of inflammation [67,177,178]. Silica also induces a degradation of the antioxidant peroxiredoxin system and suppresses the NF-κB inhibitor IκB-α [179]. Protraction of this process then results in chronic lung inflammation as well as lung fibrosis as pro-inflammatory cytokines such as IL-1, IL-18, and TNFα enhance the recruitment and proliferation of fibroblasts and mesenchymal cells to produce fibroblastic foci and to generate components of the extracellular matrix including collagen, leading to fibrotic scarring of the lung tissue [180]. As recently demonstrated, the process of silica-induced fibrosis is, besides the TLR-4/NLRP3/TGF-β signaling pathway [181] and NF-κB signaling pathways [182], also related to the activation of the acid sphingomyelin (ASM)ase/ceramide signaling pathway [183] and phosphoinositide 3-kinase (PI3K)/Akt/mTOR signaling pathway [184].

The complex pathophysiology of silicosis with the activation of multiple pro-inflammatory, pro-oxidant, and pro-fibrotic pathways indicates that besides the currently used anti-fibrotics pirfenidone and nintedanib, other approaches (anticytokines, antioxidants including NAC, and agents influencing the autophagic-lysosome system or increasing cAMP) may also be of benefit [67,185].

#### 3.5.2. NAC in In Vitro Studies of Lung Silicosis

There is a large number of in vitro studies demonstrating the positive effects of NAC for silica-exposed cells. For instance, the NAC pretreatment of silica-exposed murine macrophage cell line 264.7 lowered the TNFα mRNA and protein levels and the mRNA levels of MIP-2, MIP-1α, MIP-1β, and MCP-1 [186]. Two other studies demonstrated that the silica exposure-induced overproduction of ROS is associated with the degradation of the NF-κB inhibitor IκB-α and activation of the NF-κB pathway because a pretreatment with NAC decreased a silica-induced accumulation of ROS and inhibited the suppression of IκB-α [179], or reduced the activation of NF-κB [187]. In alveolar macrophages isolated from silica-instilled rats [188] or exposed in vitro to silica, the silica-induced elevation in intracellular ROS was associated with a decrease in intracellular GSH and cysteine and a sustained presence of apoptotic alveolar macrophages. The addition of NAC decreased the production of IL-1β and TNFα and prevented intracellular GSH depletion but did not protect the cells from apoptosis [188,189]. In another study, exposure of rat alveolar macrophages to silica induced both the inflammation and autophagy because the pretreatment with NAC decreased the expressions of TNFα and TGF-β [184].

In a recent study, NAC and desipramine, an inhibitor of ASM activity, alone or in combination, resulted in a reduction in pulmonary fibrosis, inflammation, and lipid peroxidation due to the inhibition of the Nrf2/HO-1 and ASMase/ceramide pathways, with a better effect observed for the combined treatment [190].

#### 3.5.3. NAC in Animal Studies of Lung Silicosis

In rats with intratracheally instilled silica particles, NAC treatment (500 mg/kg orally every day for 7 days before and up to 28 days after the silica administration) decreased the fibrotic score, concentrations of hydroxyproline, marker of collagen production, and MDA, a marker of oxidative stress, and prevented increases in TNFα, IL-8, and C-reactive protein [76]. In another rat model of silicosis, NAC treatment given by the gavage at a dose of 600 mg for up to 28 days reduced the formation of silicotic nodules and the contents of the mitochondrial apoptosis pathway-related proteins cytochrome C and caspases-3 and -9, decreased the fibrosis markers procollagens-I and -III, lowered the intracellular ROS, and prevented the decline in mitochondrial transmembrane potential (MTP) [77].

In a murine model of silicosis, NAC was administered by the gavage every day at three different doses for 24 h, or 1, 2, 3, 4, or 5 months. The NAC treatment suppressed the production of pro-inflammatory cytokines and MDA, with more potent effects observed for moderate and high doses of NAC. In addition, NAC improved the activities of the antioxidant markers glutathione peroxidase and SOD, elevated the total antioxidant capacity, and down-regulated the oxidizing enzymes NADPH oxidase 2, inducible NO synthase, and xanthine oxidase. Moreover, NAC decreased the fibrotic response with lower deposition of collagen and partially reversed the EMT and mesenchymal cell mobility [191].

#### 3.5.4. NAC in Clinical Studies of Lung Silicosis

Although we have found no clinical trial demonstrating the effects of NAC-only in lung silicosis, there are several studies showing advances in a combined therapy with NAC and tetrandrine, an alkaloid used in traditional Chinese medicine because of its multiple anti-inflammatory and anti-fibrotic effects. For instance, tetrandrine (60 mg–100 mg, 3 times daily for 6 days/week, for 3 months) combined with a delivery of NAC (effervescent tablet 600 mg, 1–2 times/day, for 8 months) enhanced pulmonary functions and lowered TNFα and IL-6 in the plasma of patients with silicosis [50]. In another study, the combined treatment was given as follows: NAC effervescent tablets, 600 mg, twice a day; tetrandrine tablets, 100 mg, twice a day; 6 days/week, for 8 months. The NAC-plus-tetrandrine enhanced the pulmonary functions, exercise tolerance, and occurrence of respiratory symptoms [192]. The administration regime of NAC (effervescent tablet 600 mg twice a day, with a 12-day treatment course per month in the first two months, then one course every two months, four courses in total) and tetrandrine (60–100 mg three times a day for 6 days a week, one treatment course for 3 months, then stopping for 1 month, and the second course for 3 months) alleviated the respiratory distress and enhanced exercise tolerance; decreased the plasma levels of TGF-β1, a potent fibrogenic and pro-inflammatory factor; and decreased matrix metalloproteinase (MMP)-7, a key factor in fibrosis which degrades cytoplasmic matrix components [81].

The most important in vivo studies and clinical trials on NAC use are provided in Table 1 and Table 2.

## 4. Limitations and Challenges for the Future

Considering the wide spectrum of the antioxidant and anti-inflammatory properties of NAC, one can expect convincing therapeutic effects in the diseases with inflammation and oxidative stress in their pathogenesis, e.g., COPD or asthma. However, the clinical efficacy of NAC obviously lags behind the preclinical studies. There are several possible explanations for this discrepancy. First, in vitro experiments evaluate NAC effects in the selected type of pulmonary cells isolated from the interactions with other lung or immune cells that may influence the action of NAC in in vivo conditions. Other limitations arise from the use of animal models of respiratory disorders which are not able to reproduce completely the situation in a diseased human [177,193,194,195,196,197,198]. While in animals the disease is modeled by a single instillation or repetitive exposure to the pathogen for several days/weeks, a mode of the lung injury in humans is mostly different as it includes chronic inflammatory, oxidative, and fibrotic changes in the tissue due to cumulative targeting of the lung. Moreover, for modeling the respiratory diseases, healthy young animals are used and kept in standard conditions without any concomitant diseases [177,193,194,195,196,197,198]. This is completely different from humans where the chronic lung disorders are usually detected in middle or older age and the changes are often associated with smoking, obesity, and other concomitant disorders. In addition, the effects of the delivered NAC may be influenced by inter-species differences (rat vs. human, mouse vs. human, etc.) in the response to a given therapy because of differences in the immune responses, the specifics of inflammatory and metabolic pathways, etc. Therefore, the dose of the given NAC cannot also be transferred directly from the animal model to humans without any correction.

Insufficient dosing responsible for low concentrations of NAC in the tissues may be the other factor potentially responsible for a weak response to NAC treatment in humans. Higher delivered doses may enhance the NAC bioavailability and thereby improve its effects [199], while even very high doses of NAC (up to 3000 mg/day) were demonstrated to be safe and well tolerated [15]. Because adverse effects (especially gastrointestinal symptoms) may limit the oral administration, other ways of delivery of high doses of NAC, e.g., by inhalation, can be considered [15]. However, Ehre et al. found that a limited mucolytic effect and the rapid clearance of inhaled NAC cannot be overcome by the higher dose which, in addition, caused a local inflammatory response and epithelial injury in mice [99]. Thus, an optimum balance keeping the adequate reduction of the mucus disulfide bonds and effective ciliary transport should be kept [200].

Dominance of a specific type of polymeric mucin in individual respiratory disorders has to be also considered [99]. For instance, mucin MUC5AC expression is increased and MUC5B is decreased in asthma [201], MUC5AC may elevate in the bronchiolar epithelium correlating with smoking and MUC5B may elevate in the bronchiolar lumen of patients with COPD [202], while both mucins may decrease in stable cystic fibrosis but increase in pulmonary exacerbation [203,204]. Ehre et al. demonstrated that NAC generates only a negligible reduction in MUC5B in both in vitro and in vivo conditions [99] which may partially explain the questionable results of NAC delivery in some clinical studies. This finding logically opens the door for the introduction of novel mucolytic and antioxidant agents instead of NAC in the future [99,205,206].

The level of oxidative stress may be an additional factor which may influence the therapeutic effect of NAC; thus, NAC may exert a more obvious effect in diseases with more severe oxidative stress and inflammation including the activation of the NLRP3 inflammasome such as silicosis, while in respiratory diseases with weaker oxidative and inflammatory changes the effect of NAC may be less obvious. The NLRP3 inflammasome is one of the intracellular NOD-like pattern recognition receptors which may initiate inflammatory pathways after stimulation with, e.g., lipopolysaccharide (LPS) mediated via TLR4 receptors and the activation of NF-κB or with inhaled particulate matter, including silica and cigarette smoke [207,208]. The activated inflammasome catalyzes the caspase-1 activation that leads to pyroptosis, a highly pro-inflammatory type of programed cell death, and the production of IL-1β, IL-18, and other pro-inflammatory cytokines as well as large amounts of ROS [207,208]. The inflammasome plays a role in immune responses in acute infections, including COVID-19 and influenza A [209,210,211]. Nevertheless, chronic activation of the inflammasome may also contribute to persistent inflammation and fibrotic responses in the lung [208]. The contribution of the NLRP3 inflammasome has been largely demonstrated in fibrotic lung diseases, including silicosis [67,212,213,214] and cystic fibrosis [215,216], but the role of the NLRP3 inflammasome is increasingly discussed in the pathogenesis of COPD or asthma, too [208,217,218]. NAC has the potential to reverse the activation of the NLRP3 inflammasome as shown in, e.g., LPS-exposed H9C2 cardiomyocytes [219], in macrophages exposed to carbon nanotubes and silica [220], in bisphenol S-induced oxidative stress in murine RAW264.7 cells [221], or in cells infected by SARS-CoV-2 [222], thereby representing additional therapeutic targets for NAC.

NAC can work very effectively as a drug for paracetamol poisoning in clinical settings, not just in clinical trials. On the contrary, in chronic diseases associated with antioxidant deficiency or imbalance, or during administration to otherwise healthy elderly patients as dietary supplements, there is no consistency in support of antioxidant capacity and efficacy. For instance, a recent meta-analysis of data from healthy males showed a significant improvement in exercise performance, antioxidant capacity, and glutathione homeostasis [223]; however, the clinical trial by Paschalis et al. [224] revealed that NAC supplementation seems to be more effective in humans with low levels of glutathione, while a lack of effect may be observed in humans with moderate and high glutathione levels. This can be associated with several reasons, e.g., a shorter duration of treatment and faster observation of results in paracetamol poisoning, contrary to long-term and mild effects in COPD or other chronic respiratory diseases, or a biphasic and context-dependent response of cells on the pleiotropic action of NAC [225].

Despite the fact that NAC was first approved by the FDA as a respiratory drug in 1963, and since then it has been also used to prevent liver damage from paracetamol overdose and poisoning, it has been available in dietary supplement products for decades and NAC is considered safe for most people. However, the FDA claims that there is no evidence that NAC was used as a supplement prior to its use as a drug. Therefore, including NAC in a supplement makes the product an unapproved drug and thus illegal. Nevertheless, in August 2022, the FDA announced some steps to allow the use of NAC as a dietary supplement (i.e., to provide by regulation that NAC is not excluded from the definition of dietary supplement). If, among other considerations, the FDA does not identify safety-related concerns during the review of the available data and information, they are likely to propose a rule providing that NAC is not excluded from the definition of dietary supplement [226].

## 5. Conclusions

NAC is a drug with a wide spectrum of actions. Thanks to its antioxidant and anti-inflammatory properties, negligible adverse effects even at high doses compared to other anti-inflammatory drugs, and low price, the spectrum of indications of NAC expands to other respiratory diseases besides cystic fibrosis. Nevertheless, there have arisen several challenges for the future use of NAC, such as optimization of the dosing for oral and inhaled administration or searching for novel formulas with improved bioavailability.

## Figures and Tables

**Table 1:** In vivo studies demonstrating effects of NAC in animal models of cystic fibrosis (CF), chronic obstructive pulmonary disease (COPD), bronchial asthma, idiopathic pulmonary fibrosis (IPF), and lung silicosis.

Animal Model	Species	NAC Dose/Way of Delivery	Major Findings	Study
Model of CF	WT and βENaC-overexpressing mice	NAC (15 μL, 50 mg/kg), i.t. instillation in WT mice; NAC (200 mM, nasal nebulizations) in βENaC-overexpressing mice	WT mice: no reduction in Muc5b, rapid NAC clearance, ↑ total cells and neutrophils in BALF, epithelial injury; βENaC-overexpressing mice: no reduction in AW mucus obstruction	[99]
Cigarette smoke (CS)-induced model of COPD	Sprague Dawley rats	NAC (800 mg/kg, once a day, i.g.), from 30 days of CS exposure	↓ lung damage, emphysema, and alveolar septal cell apoptosis by partial reversing of ↓ VEGF	[118]
Ozone-induced model of COPD	C57/BL6 mice	NAC (100 mg/kg, i.p.) before each exposure (preventive) or after completion of exposure (therapeutic), for 6 weeks	Preventive NAC: ↓ BALF macrophages, ↓ AW smooth muscle mass. Therapeutic NAC: reversed AW hyperresponsiveness, ↓ AW smooth muscle mass and apoptotic cells	[119]
Cigarette smoke (CS)-induced model of COPD	Rats	NAC (800 mg/kg, once a day, i.g.), from 2 days before the model establishment	↓ inflammation, enhanced lung functions, ↓ fibrotic changes	[120]
OVA-induced model of asthma	Norway rats	NAC (3 mM/kg, once a day, p.o.), pretreatment daily from 7 days before challenge	↓ AW hyperresponsiveness, ↓ lipid peroxidation, ↓ oxidized GSH, ↓ TNFα, iNOS, ICAM-1, and MUC5AC	[138]
OVA-induced model of asthma	Balb/c mice	NAC (320 mg/kg, i.p.), 30 min before and 12 h after each challenge	↓ AW hyperresponsiveness, ↓ neutrophils and eosinophils in BALF, ↓ IL-13 and IL-5	[139]
OVA-induced model of asthma	Balb/cJ mice	NAC (15 mg/100 g body weight, i.n.), 45 min before three i.n. challenges	↓ AW inflammation, ↓ mitochondrial damage, ↓ goblet cell hyperplasia, ↓ eosinophil extracellular traps, ↓ oxidative stress in lungs	[140]
OVA-induced model of asthma	Balb/c mice	NAC (100 mg/kg), inhalation 1 h after each challenge for 6 days	↓ AW hyperresponsiveness, ↓ AW inflammation, ↓ claudin 18	[141]
Obesity-associated model of asthma	C57BL/6 J mice	NAC (200 mg/kg, i.g.)	↓ inflammation and oxidative stress, ↓ IKK-β and NF-κB-P65, ↑ IκB-α, ↓ MDA	[142]
Chemical-induced model of asthma	Balb/c mice	NAC (100 mg/kg, i.p.), after each challenge	↓ AW hyperresponsiveness, ↓ AW inflammation, ↓ goblet cell metaplasia, ↓ inflammatory cell counts in BALF, ↓ IL-4 and IL-5, ↓ oxidative stress	[143]
Bleomycin-induced model of IPF	Sprague Dawley rats	NAC (3 mM/kg, p.o.), daily from 1 week prior to i.t. bleomycin instillation and for 14 d postinstillation	↓ collagen and ↓ inflammatory cells in the lungs, ↑ total GSH and taurine in BALF, no effect on lung edema formation	[72]
Bleomycin-induced model of IPF	Sprague Dawley rats	NAC (3 mM/kg, p.o.), daily from 1 week prior to i.t. bleomycin instillation and for 14 d postinstillation	↓ collagen and fibrotic area in the lungs, ↓ MUC5ac expression, ↓ TNFα and MPO, ↓ mucus hypersecretion	[73]
Bleomycin-induced model of IPF	Sprague Dawley rats	NAC (3 mM/kg, p.o.), daily from 1 week prior to i.t. bleomycin instillation and for 14 d postinstillation	↓ hydroxyproline, ↓ depletion of GSH peroxidase, prevention of ↑ in MPO, NO, and MDA	[161]
Bleomycin-induced model of IPF	Sprague Dawley rats	NAC (490 mg/kg/day, p.o.), daily from bleomycin instillation	↓ lysyl oxidase activity, ↑ GSH, ↓ lung fibrosis, ↓ TGF-β1 and α-SMA expression	[75]
Bleomycin-induced model of IPF	Sprague Dawley rats	NAC (300 mg/kg/day, i.p.), daily from 1 week prior to i.t. bleomycin instillation and for 15 d postinstillation	↑ GSH/GSSG ratio, ↓ NO, ↓ lipid hydroperoxides, ↓ lung weight, ↓ hydroxyproline, ↓ inflammatory cytokines, MPO, and LDH, ↓ deposition of collagen	[74]
Bleomycin-induced model of IPF	CF1 mice	NAC (20 mg/kg/day, i.p.), from day 0 of bleomycin instillation, for 60 days	↓ lung injury, ↓ LDH, neutrophils, total cell counts, and protein in BALF	[162]
Bleomycin-induced model of IPF	Wistar rats	NAC (250 mg/kg/day, i.g.), from day 0 of bleomycin instillation, for 31 days	↓ lung inflammation and fibrosis, ↓ MDA, ↑ SOD	[163]
Bleomycin-induced model of IPF	Wistar rats	NAC (4 mg/kg, 3 times/day, i.g.), from 1 day after bleomycin instillation, for 45 days	↓ lung inflammation and fibrosis, ↓ TGF-β1, TNFα and PDGF expressions, ↑ caveolin-1	[164]
Model of silicosis	Wistar rats	NAC (500 mg/kg/day, p.o.), for 7 days before and up to 28 days after silica instillation	↓ fibrosis score, ↓ hydroxyproline, MDA, TNFα, IL-8, and hsCRP	[76]
Model of silicosis	Sprague Dawley rats	NAC (600 mg/kg/day, by gavage), from day 0 of silica instillation, for 28 days	↓ ROS, ↓ changes in mitochondrial transmembrane potential, ↓ fibrotic changes, ↓ markers of apoptosis	[77]
Model of silicosis	C57BL/6J mice	NAC (1.73 mg/20 g, 3.46 mg/20 g, or 5.19 mg/20 g, by gavage), from day 0 of silica instillation, observation for 5 months	↓ lung injury, fibrosis, and inflammation, ↓ MDA, ↑ GSH peroxidase, SOD, total antioxidant activity, and E-cadherin, ↓ oxidizing enzymes, vimentin, and cytochrome C	[191]

Abbreviations: AW: airway, BALF: bronchoalveolar lavage fluid, CF: cystic fibrosis, COPD: chronic obstructive pulmonary disease, GSH: glutathione, GSSG: oxidized glutathione, hsCRP: high-sensitivity C-reactive protein, ICAM-1: intercellular adhesion molecule-1, i.g.: intragastric administration, IκB-α: inhibitor kappa B-α, IKK-β: inhibitor kappa B kinase-β, IL: interleukin, i.n.: intranasal administration, iNOS: inducible nitric oxide synthase, i.p.: intraperitoneal administration, IPF: idiopathic pulmonary fibrosis, LDH: lactate dehydrogenase, MDA: malondialdehyde, MPO: myeloperoxidase, MUC5ac: mucin MUC5ac, NAC: *N*-acetylcysteine, NF-κB-P65: nuclear factor-κB-P65, NO: nitric oxide, PDGF: platelet-derived growth factor, p.o.: oral administration, ROS: reactive oxygen species, α-SMA: alpha-smooth muscle actin, SOD: superoxide dismutase, TGF-β1: transforming growth factor beta 1, TNFα: tumor necrosis factor alpha, VEGF: vascular endothelial growth factor, WT: wild-type mice, ↑: increased, ↓: decreased.

**Table 2:** Clinical trials demonstrating effects of NAC in cystic fibrosis (CF), chronic obstructive pulmonary disease (COPD), bronchial asthma, and idiopathic pulmonary fibrosis (IPF).

Diagnosis	No. of Patients	Treatment	Outcomes in NAC-Treated Patients	Study
CF	21 in total (11 low-dose NAC, 10 high-dose NAC)	Oral NAC (700 mg daily or 2800 mg daily p.o.), for 12 weeks	High-dose NAC well-tolerated, trend to ↑ extracellular GSH	[100]
CF	22 in total (10 in study 1, 12 in study 2)	Inhalational NAC: Study 1: from 2 (4 mg) to 8 puffs (16 mg) of NAC, or 12 puffs (24 mg) of NAC for 5 days; Study 2: single dose 12 puffs, 24 h monitoring	Study 1: dose-dependent ↓ in sputum viscoelasticity, ↓ in sputum solids; Study 2: ↓ mucus rigidity, max. at 4 h	[101]
CF	21 in total (11 NAC, 10 control)	Oral NAC (2400 mg/day in 2 doses), for 30 days	↓ oxidized vit. C, ↑ levels of vit. C, slight improvement in lung function	[102]
CF	70 in total (36 NAC, 34 placebo)	Oral NAC (900 mg, 3 times a day) for 24 weeks	Stable lung function, but no effect on neutrophilic inflammation	[103]
CF	5 in total	Inhalational NAC (20%, 1.27 M), 90 min observation	No effect on mucus properties	[99]
COPD	50 in total (25 NAC, 25 placebo) (ISRCTN21676344)	Oral NAC (600 mg, twice daily), for 7 days (addition to corticosteroids and bronchodilators)	No effect on lung functions or median length of stay in hospital	[121]
COPD	523 in total (256 NAC, 267 placebo) (BRONCUS trial)	Oral NAC (600 mg/day), patients followed for 3 years	No prevention of deterioration in lung functions, no prevention of exacerbations	[122]
COPD	24 in total (12 NAC, 12 placebo) (NCT00476736)	Oral NAC (1200 mg/day), for 6 weeks	Beneficial effect on physical performance	[123]
COPD	120 in total (58 NAC, 62 placebo) (HIACE trial, NCT01136239)	Oral NAC (600 mg bid or placebo after 4-week run-in), for 1 year	Enhanced small AW function, ↓ exacerbation frequency	[7]
COPD	1006 in total (504 NAC, 502 placebo) (PANTHEON trial, ChiCTR-TRC-09000460)	Oral NAC (600 mg, twice daily), for 1 year	Prevention of acute exacerbations	[8]
COPD/chronic bronchitis	45 in total (23 NAC, 22 placebo) (NCT01599884)	Oral NAC (1800 mg twice daily), for 8 weeks	No clinical benefit, study prematurely terminated	[20]
COPD	100 in total (50 NAC, 50 placebo)	Oral NAC (900 mg twice daily), for 3 months	Absence of clinical improvement	[124]
COPD	121 in total (60 NAC, 61 control)	Oral NAC (0.2 g × 10 bags, 0.4 g each time, 3 times/day), for 6 months	↑ proportion of Treg in CD4+ T cells and ↓ Th17/Treg ratio	[125]
Asthma	25 in total	Oral NAC (200 mg, 3 times daily), for 9 weeks	No effect on spirometric, lung mechanics or gas exchange variables, or frequency of clinical symptoms	[144]
Asthma	50 in total (25 NAC, 25 placebo)	Oral NAC (600 mg/day), for 5 days	No effect on clinical symptoms	[145]
IPF	14 in total (8 NAC, 6 controls)	Intravenous NAC (600 mg, 1800 mg and 4800 mg), in weekly intervals	↑ GSH in low and moderate dose in IPF patients, but no effect on GSH in controls, no adverse effects	[25]
IPF	31 in total (17 NAC, 14 controls)	Oral NAC (600 mg, 3 times/day), for 5 days	↑ GSH in BALF/ELF, treatment well-tolerated	[166]
IPF	18 in total	Oral NAC (600 mg, 3 times/daily), for 12 weeks	↑ GSH in BALF/ELF, ↓ methionine sulfoxide content of BALF proteins, improved lung functions	[167]
IPF	76 in total (38 NAC, 38 controls)	Inhalational NAC (352.4 mg, twice daily), observation for 48 weeks	No differences in lung functions	[168]
IPF	28 in total	Inhalational NAC (352.4 mg, twice daily), observation for 26 weeks	Prevented a ↓ in FVC in mild-to moderate IPF, better effect in more severe IPF	[169]
IPF	22 in total	Inhalational NAC (352.4 mg, twice daily), observation for 12 months	Prevented ↓ in FVC, ↓ oxidative imbalance, better effect in more severe oxidative stress	[170]

Abbreviations: AW: airway, BALF: bronchoalveolar lavage fluid, CF: cystic fibrosis, COPD: chronic obstructive pulmonary disease, ELF: epithelial lining fluid, FVC: forced vital capacity, GSH: glutathione, IPF: idiopathic pulmonary fibrosis, NAC: *N*-acetylcysteine, ↑: increased, ↓: decreased.

## Data Availability

Data is contained within the article.
